# Deep medullary veins disruption in cerebral small vessel disease: links to AI-quantified lesions and cognitive decline

**DOI:** 10.3389/fneur.2025.1647684

**Published:** 2025-10-20

**Authors:** Yong-Lin Liu, Han-Peng Yin, Feng Wang, Ting-Yu Bao, Min-Yi Yao, Yi-Mian Yuan, Man-Qiu Liang, Fang Wang, Yang-Kun Chen, Li-An Huang

**Affiliations:** ^1^The First Affiliated Hospital, Jinan University, Guangzhou, Guangdong, China; ^2^Department of Neurology, The Tenth Affiliated Hospital, Southern Medical University (Dongguan People's Hospital), Dongguan, Guangdong, China; ^3^Intelligent Brain Imaging and Brain Function Laboratory (Dongguan Key Laboratory), Dongguan People's Hospital, Dongguan, Guangdong, China; ^4^Department of Radiology, The Tenth Affiliated Hospital, Southern Medical University (Dongguan People's Hospital), Dongguan, Guangdong, China

**Keywords:** cerebral small vessel disease, deep medullary veins, white matter hyperintensities, cognitive impairment, artificial intelligence neuroimaging

## Abstract

**Background:**

The relationship between cerebral small vessel diseases (CSVD) and deep medullary veins (DMV) has been the subject of only a limited number of studies to date, with the majority of research focusing on the correlation between DMV and cognitive function in patients with CSVD. The present study aims to quantitatively evaluate the relationship between DMV and CSVD imaging markers, utilizing an artificial intelligence neuroimaging system to achieve this objective. Furthermore, an investigation was conducted into the correlation between DMV disruption and cognitive impairment.

**Methods:**

This cross-sectional study enrolled patients with CSVD imaging markers who were admitted to the Department of Neurology at our hospital. The quantitative assessment of CSVD imaging markers, encompassing white matter hyperintensities (WMH), lacunar infarction (LI), and cerebral microbleeds (CMB), was conducted employing the AccuBrain^®^ neuroimaging system. The DMV was evaluated using susceptibility-weighted imaging (SWI) with a semi-quantitative scale. The Montreal Cognitive Assessment (MoCA) score was evaluated for each patient.

**Results:**

The study’s sample population comprised 171 patients. Multivariate ordinal logistic regressions indicated that DMV score was significantly associated with WMH volume, LI count, and CMB count (*p* < 0.05). In the context of multivariate linear regression analysis, a significant negative correlation was observed between the DMV score and the MoCA score, with the latter being adjusted for confounding variables such as age, education, gender, and smoking history (*p* = 0.003). The application of path analysis revealed a significant correlation between the DMV score and the MoCA score, which indicates that WMH volume plays a mediating role in this relationship, thereby offering a novel perspective on cognitive function and neurodegenerative processes.

**Conclusion:**

DMV score is associated with the severity of WMH, CMB, and LI, as well as cognitive performance decline, implicating that cerebral venule damage may play a role in the development of CSVD and related cognitive impairment.

## 1 Introduction

Cerebral small vessel disease (CSVD) is a prevalent form of cerebrovascular disease, primarily triggered by pathological alterations in small penetrating arteries, capillaries, and veins within the brain (1). It has been documented that approximately 5% of individuals over the age of 50 and nearly 100% of those over 90 years of age are affected by CSVD (2). CSVD has been determined to be a causative agent in approximately 25% of ischemic strokes in individuals over the age of 65 (3). Furthermore, moderate-to-severe CSVD is associated with a significantly increased risk of recurrent stroke (4). Besides, CSVD has been demonstrated to result in cognitive impairment. A previous study revealed that CSVD-related dementia accounted for 45% of all causes of dementia (5). Moreover, CSVD has been associated with various health concerns, including but not limited to, gait disorders (6), mood disorders (7), and sleep disorders (8). It is challenging to envision these minute vessels on conventional magnetic resonance imaging (MRI) scans. It is widely acknowledged that imaging markers on MRI, such as white matter hyperintensities (WMH), lacunar infarction (LI), cerebral microbleeds (CMB), enlarged perivascular space, and brain atrophy, can effectively reflect the severity of CSVD in the brain (3).

CSVD encompasses a range of etiological types, including arteriosclerosis/age-related, amyloidosis-related, and inflammation/immune-mediated forms. Among these, arteriosclerosis/age-related CSVD is the most prevalent (9). The etiology of CSVD remains to be elucidated. Preliminary research indicates that the primary pathological alterations associated with CSVD encompass abnormalities in small arteries, including arteriosclerosis, lipid hyaline degeneration, and fibrinoid necrosis (44). These abnormalities predominantly manifest in the context of arteriosclerosis/age-related CSVD, which bears risk factors analogous to those observed in systemic vascular diseases, such as advanced age, diabetes, and hypertension (10). Although arteriolar changes are the primary pathological changes in CSVD, preliminary evidence suggests that cerebral veins also play an important role in balancing and stabilizing cerebral blood flow, and changes in cerebral veins may also trigger or exacerbate CSVD (11, 12). DMV are diminutive veins situated in the white matter encompassing the ventricles of the brain, with diameters ranging from tens to hundreds of micrometers. These veins are responsible for the drainage of cerebral venous blood, ultimately connecting to the deep venous system (13). It has been determined that the DMV can be identified through susceptibility-weighted imaging (SWI) of 3.0-tesla (T) MRI (14). A previous study demonstrated that the alteration in continuity or the reduction in the number of DMV exhibited on SWI can be regarded as the imaging manifestation of venous collagenosis (VC), which is believed to be associated with CSVD (15). A number of earlier studies have indicated a correlation between the impairment of the DMV and the severity of WMH (16, 17), as well as the augmented number of LI (16) and CMB (18). However, the limitations of these studies stem from their relatively small sample size, which necessitated the use of semi-quantitative or visual measurements to assess the CSVD imaging markers. This methodological choice severely restricted the ability to quantitatively determine the volume of WMH. The objective of this study was to investigate the association between DMV visualization on SWI and quantitatively measured CSVD markers, including WMH, LI, and CMB. This investigation was conducted using an artificial intelligence (AI)-based neuroimaging system. In addition, the objective was to assess the correlation between structural disruptions in the DMV and the severity of cognitive impairment in this particular sample.

## 2 Methods

### 2.1 Study participants

We prospectively recruited CSVD patients who were admitted in the Department of Neurology in Dongguan People’s Hospital from 1 June 2021 to 30 December 2022. The study cohort comprised patients admitted for neurological manifestations of CSVD, encompassing cognitive decline, dizziness, gait disturbance, minor sensorimotor deficits, or a history of transient ischemic attack. A subset of patients was admitted for management of vascular risk factors (e.g., hypertension, diabetes) and underwent neuroimaging in the absence of acute symptoms. All patients underwent a standardized brain MRI as part of their clinical evaluation. The inclusion criteria were as follows: (1) age≥18 years, (2) CSVD imaging features on brain MRI, including: ① WMH with Fazekas score≥2; or ② Fazekas score = 1 combined with ≥ 2 vascular risk factors (including hypertension, type 2 diabetes, lipoprotein metabolism disorders, obesity and smoking); or ③ Fazekas score = 1 with LI; or ④ Recent small subcortical infarction (19), (3) undergone relevant MRI protocols, including T1-weighted imaging (T1WI), T2-weighted imaging (T2WI), fluid-attenuated inversion recovery (FLAIR), diffusion-weighted imaging (DWI), SWI, and MR angiography (MRA), and (4) willing to sign the informed consent. Patients were excluded based on the following criteria: (1) a history of acute cerebral infarction within the past 3 months; (2) a history of intracranial hemorrhage; (3) a history of massive ischemic stroke (IS); (4) one or more IS lesions surrounding the ventricles; (5) other central nervous system diseases, including: tumors, trauma, degenerative, toxic, infectious, immune demyelinating or metabolic diseases; (6) moderate–severe stenosis or occlusion of intracranial or extracranial large arteries demonstrated on MRA; and (7) confirmed hereditary CSVD. This study was approved by the hospital ethics committee (approval number: KYKT2021-049-A1). All subjects provided informed consent in accordance with the Declaration of Helsinki.

### 2.2 Clinical data collection

A comprehensive array of clinical data was meticulously collected for each case, encompassing demographic information such as age and sex, key risk factors associated with cerebrovascular diseases, namely hypertension, diabetes, and smoking history. The dataset also included detailed hematologic findings, including markers of renal function and metabolism such as serum creatinine, uric acid, along with lipid profiles, homocysteine (Hcy), and hemoglobin A1c (HbA1c). The Montreal Cognitive Assessment (MoCA) was utilized to evaluate the patients’ cognitive function. To ensure the consistency of the MoCA score results, the MoCA scores for all cases were completed by an experienced attending neurologist (Han-peng Yin).

### 2.3 Imaging protocol

A brain MRI was performed on each patient, encompassing three-dimensional T1WI (3D-T1WI), T1WI, T2WI, FLAIR, DWI, SWI, and three-dimensional time-of-flight MRA (3D-TOF-MRA). All scanning was carried out with a 3.0 T system (Skyra, Siemens Medical, Erlangen, Germany). The parameters for each sequence are enumerated in Table 1.

**Table 1:** Parameters for each sequence of MRI scanning in this study.

	TR (ms)	TE (ms)	FOV (mm2/mm3)	ST/gap (mm)	TA(s)	Matrix
3D-T1WI	2,200	2.26	256 × 256	1/0.5	222	320 × 256
T1WI	1,500	11	220 × 185	4/1.2	86	320 × 256
T2WI	5,100	96	220 × 199	4/1.2	146	320 × 256
FLAIR	9,000	94	230 × 187	4/1.2	128	320 × 256
SWI	30	20	220 × 195	1/0.2	332	512 × 512
MRA	21	3.42	200 × 160 × 160	0.7/−0.14	226	384 × 320

3D-T1WI, Three-dimensional T1-weighted imaging; FLAIR, fluid-attenuated inversion recovery; FOV, field of view; MRA, magnetic resonance angiography; MRI, magnetic resonance imaging; ST, slice thickness; SWI, susceptibility-weighted imaging; TA, time of acquisition; TE, time of echo; TR, time of repetition; T1WI, T1-weighted imaging; T2WI, T2-weighted imaging.

### 2.4 Measurement of DMV

In accordance with the findings of a preceding study, the DMV was evaluated by means of a comprehensive analysis of five successive periventricular slices derived from SWI phase images. This analysis commenced at the level of the ventricles immediately above the basal ganglia and proceeded until the point at which the ventricles became undetectable. This approach was applied to each patient. The bilateral brain regions that contained the DMV were segmented into six distinct segments, including the frontal, parietal, and occipital segments. In order to perform semi-quantitative assessments of the DMV, each segment was independently scored based on its continuity and visibility. DMV scores ranged from 0 to 3. Therefore, the overall DMV score ranged from 0 to 18. A higher DMV score indicated a less prominent DMV that could be observed, and a score of 18 indicated no obvious DMV was visible (16). Two neuroradiologists specializing in MRI (Man-Qiu Liang and Feng Wang) who were unaware of the patients’ clinical information independently evaluated the imaging and assigned a DMV score. Subsequent to their individual observations, the two observers reviewed all of the images to reach a final inter-observer consensus.

### 2.5 Measurement of WMH, LI, and CMB

**Figure 1:**
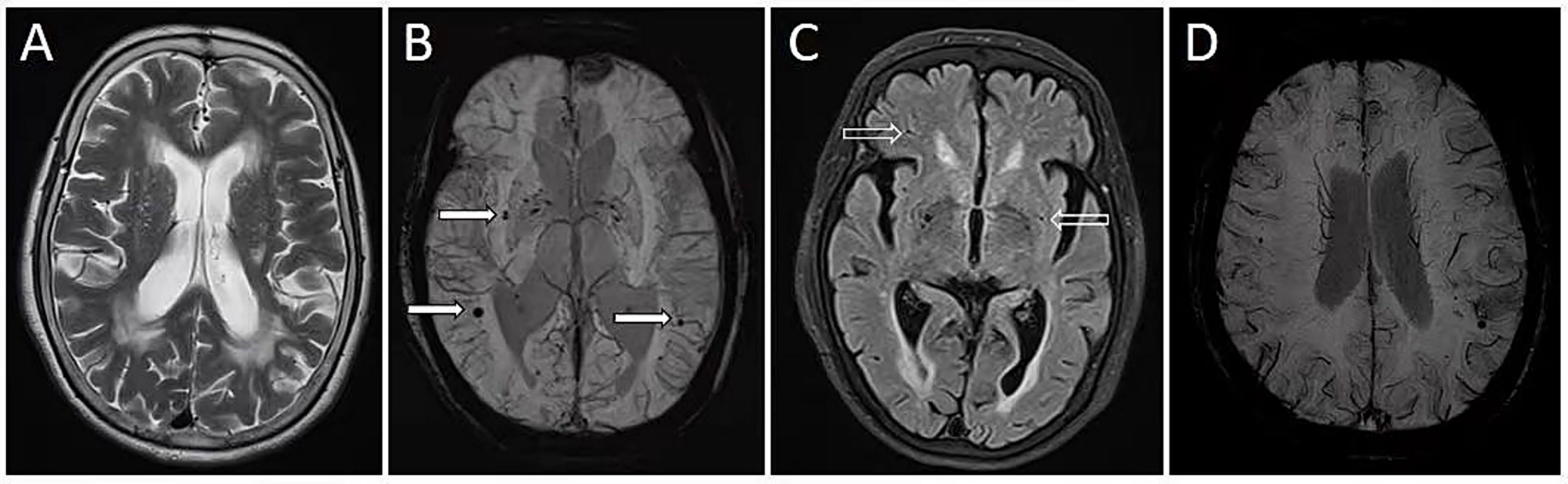
Quantitative neuroimaging markers in a hypertensive patient with cognitive decline. A 71-year-old woman with a history of hypertension was admitted due to cognitive decline. She received a score of 8 on the Montreal Cognitive Assessment. Magnetic resonance imaging (MRI) revealed severe white matter intensities (WMH) **(A)**, cerebral microbleeds (CMB) (**B**, solid arrows), and lacunar infarctions (LI) (**C**, hollow arrows). Quantitative analysis using the AccuBrain@ software revealed a WMH volume of 59.7 mL, CMB count of 23, and LI count of 17. MRI susceptibility-weighted imaging also identified discontinuous deep medullary veins (DMV) **(D)**, with a DMV score of 15.

The MRI features of WMH, LI, and CMB were then quantified using AccuBrain@ (BrainNow, Shenzhen, China) (45–47). The segmentation and volume quantification of the WMH were performed using a coarse-to-fine mathematical morphology and calculated automatically. The CMB counting on SWI was implemented by a fully connected network that was trained on a large number of SWI MRIs with their manually identified CMB labels. The algorithm will generate a probability map, indicating the likelihood of a position with CMB. This probability map is subsequently thresholded by a cutoff value that is determined during the training process. The number of CMB was documented. LI was segmented in T2 FLAIR images. A series of pre-processing steps were performed initially, including skull stripping, intensity normalization, and contrast enhancement. Subsequently, the post-processed images were segmented using a convolutional neural network, with the segmentation process guided by a set of manually annotated labels numbering in the hundreds. Consequently, the characterization of WMH was performed in terms of volume, while LI and CMB were quantified in terms of number. The typical case is illustrated in Figure 1.

### 2.6 Statistical analysis

Statistical analyses were conducted using SPSS for Windows (Version 24.0, IBM Corp., Armonk, NY, United States). Continuous variables with a normal distribution were reported as mean ± standard deviation (SD), while non-normally distributed variables were reported as median and interquartile range (IQR), and categorical variables were described as number (%). The primary objective of this study was to investigate the relationship between DMV score and CSVD imaging markers, including WMH, CMB, and LI, as well as the relationship between DMV score and MoCA score. Due to the non-normal distribution of WMH volume and the numbers of LI and CMB, these variables were divided into four classes by IQR and converted into ordered categorical variables. A univariate ordinal regression analysis was performed, and variables with a *p*-value less than 0.05 were entered into a multivariate ordinal regression analysis. As the MoCA score is a normally distributed variable (confirmed by one-sample Kolmogorov–Smirnov test), a univariate linear regression analysis was conducted, and variables with a *p*-value less than 0.05 were entered into a multivariate linear regression analysis. SPSS AMOS 21.0 was utilized to build a structural equation model (SEM) and analyze the impact of various influencing factors on the decline of MoCA score. The maximum-likelihood was applied to estimate the parameters of SEM. The chi-square degrees of freedom (χ^2^/df), goodness-of-fit index (GFI), and root mean square error of approximation (RMSEA) are used as indicators to measure the fit of a model’s structure. The iteration ends after 5,000 times. A model was considered to have a satisfactory fit if the χ2/df value was less than 3, the RMSEA was below 0.07, and GFI exceeded 0.9, as per established criteria (20). Statistical significance was defined as *p* < 0.05 (two-sided).

## 3 Results

During the study period, 206 consecutive patients met the inclusion criteria. However, 35 patients were subsequently excluded due to various reasons, including presence of asymptomatic acute ischemic stroke (IS) lesions confirmed by MRI (*n* = 8), one or more IS lesions surrounding the ventricles (*n* = 13), probable Alzheimer’s disease (*n* = 5), and moderate–severe stenosis of intracranial large arteries demonstrated on MRA (*n* = 9). Therefore, a total of 171 patients were ultimately included in our study. The flowchart of this study is presented in Supplementary Figure 1. The average age of the 171 patients was 65.9 ± 10.1 years, with 77 (45.0%) patients being male. The demographic and clinical characteristics of this study are demonstrated on Table 2.

**Table 2:** Demographic and clinical characteristics of the study cohort.

Characteristics	Mean(SD)/Median(IQR)/n(%) *n* = 171
Age (years)	65.9 (10.1)
Men (*n*, %)	77 (45.0%)
Education (*n*, %)	
Illiteracy	31 (18.1%)
Primary school	67 (39.2%)
Junior high school	42 (24.6%)
High school	19 (11.1%)
College	12 (7.0%)
Hypertension (*n*, %)	121 (70.8%)
Diabetes (*n*, %)	52 (30.4%)
Smokers/ex-smokers (*n*, %)	33 (19.3%)
Serum creatinine (μmol/L)	70.9 (24.9)
Uric Acid (μmol/L)	366.2 (102.8)
TCH (mmol/L)	5.0 (1.7)
LDL-C (mmol/L)	3.1 (0.9)
Hcy (mmol/L)	13.4 (3.9)
HbA1c (%)	5.9 (5.6, 6.3)
DMV score	4.0 (2.0, 8.0)
WMH volume (mL^3^)	4.7 (2.1, 12.2)
CMB number	0.0 (0.0, 2.0)
LI number	7.0 (4.0, 10.0)
MoCA	
Illiteracy	10.29 (3.98)
Primary school	15.66 (5.50)
Junior high school	18.19 (5.10)
High school	20.72 (3.88)
College	25.17 (3.21)

CMB, cerebral microbleed; DMV, deep medullary vein; HbA1c, glycated hemoglobin, type A1c; Hcy, homocysteine; IQR, interquartile range; LDL-C, low density lipoprotein cholesterin; LI, lacunar infarction; MoCA, Montreal Cognitive Assessment; SD, standard deviation; WMH, white matter hyperintensities.

### 3.1 Univariable analysis

Univariable ordinal logistic regression analyses were performed to investigate the relationship between DMV score and WMH, CMB as well as LI, respectively. Higher WMH volume was associated with higher DMV scores and serum creatinine (SCR) levels (*p* < 0.05). Patients with a higher number of CMBs or LIs had significantly higher DMV scores (*p* < 0.05). A univariable linear regression analysis was conducted to examine the relationship between DMV score and MoCA score. Patients with lower MoCA scores were generally older, had lower level of education and higher DMV scores, and were more likely to be male (*p* < 0.05). Conversely, they were less likely to have a history of smoking (*p* < 0.05). The results of all univariable analysis were presented in Supplementary Tables 1–4.

### 3.2 Multivariable analysis

Multivariate ordinal logistic regression analysis was conducted subsequent to univariate ordinal logistic regression analysis. Variables with a *p*-value less than 0.05 in the univariable analysis were included. In the analysis of the association between risk factors and WMH, CMB number and LI number, DMV score demonstrated significant association with WMH (*P <* 0.001), CMB number (*p* = 0.014) and LI number (*p* = 0.001). Since age, hypertension, and diabetes are widely-accepted as risk factors for CSVD (10), they were incorporated into the regression analysis to investigate the association between risk factors and WMH volume, CMB, and LI. After adjusting for age, hypertension and diabetes, DMV score maintained a significant correlation with WMH volume as well as the number of CMB and LI. The results of the multivariate ordinal logistic regressions analysis examining the association between risk factors and CSVD markers, were tabulated in Table 3.

**Table 3:** Multivariate ordinal logistic regressions analysis of the association between risk factors and CSVD markers.

Characteristics	WMH volume		CMB number		LI number	
	OR(95%CI)	*P*	OR(95%CI)	*P*	OR(95%CI)	*P*
Model 1						
SCR	1.018 (1.004–1.033)	0.013	–	–	–	–
DMV score	1.329 (1.210–1.459)	0.000	1.119 (1.023–1.224)	0.014	1.141 (1.054–1.234)	0.001
Model 2*						
Age	1.000 (0.971–1.031)	0.978	0.955 (0.919–0.993)	0.020	0.968 (0.938–0.998)	0.035
Hypertension	1.151 (0.606–2.185)	0.668	1.136 (0.547–2.362)	0.732	0.795 (0.429–1.474)	0.467
Diabetes	0.487 (0.254–0.934)	0.030	0.983 (0.476–2.027)	0.962	0.985 (0.534–1.814)	0.960
SCR	1.019 (1.004–1.034)	0.013	–	–	–	–
DMV score	1.341 (1.215–1.479)	0.000	1.173 (1.061–1.297)	0.002	1.184 (1.087–1.289)	0.000

CI, confidence interval; CMB, cerebral microbleed; CSVD, cerebral small vessel disease; DMV, deep medullary vein; LI, lacunar infarction; SCR, serum creatinine; OR, odds ratio; WMH, white matter hyperintensities. *Adjusted for age, hypertension and diabetes.

**Figure 2:**
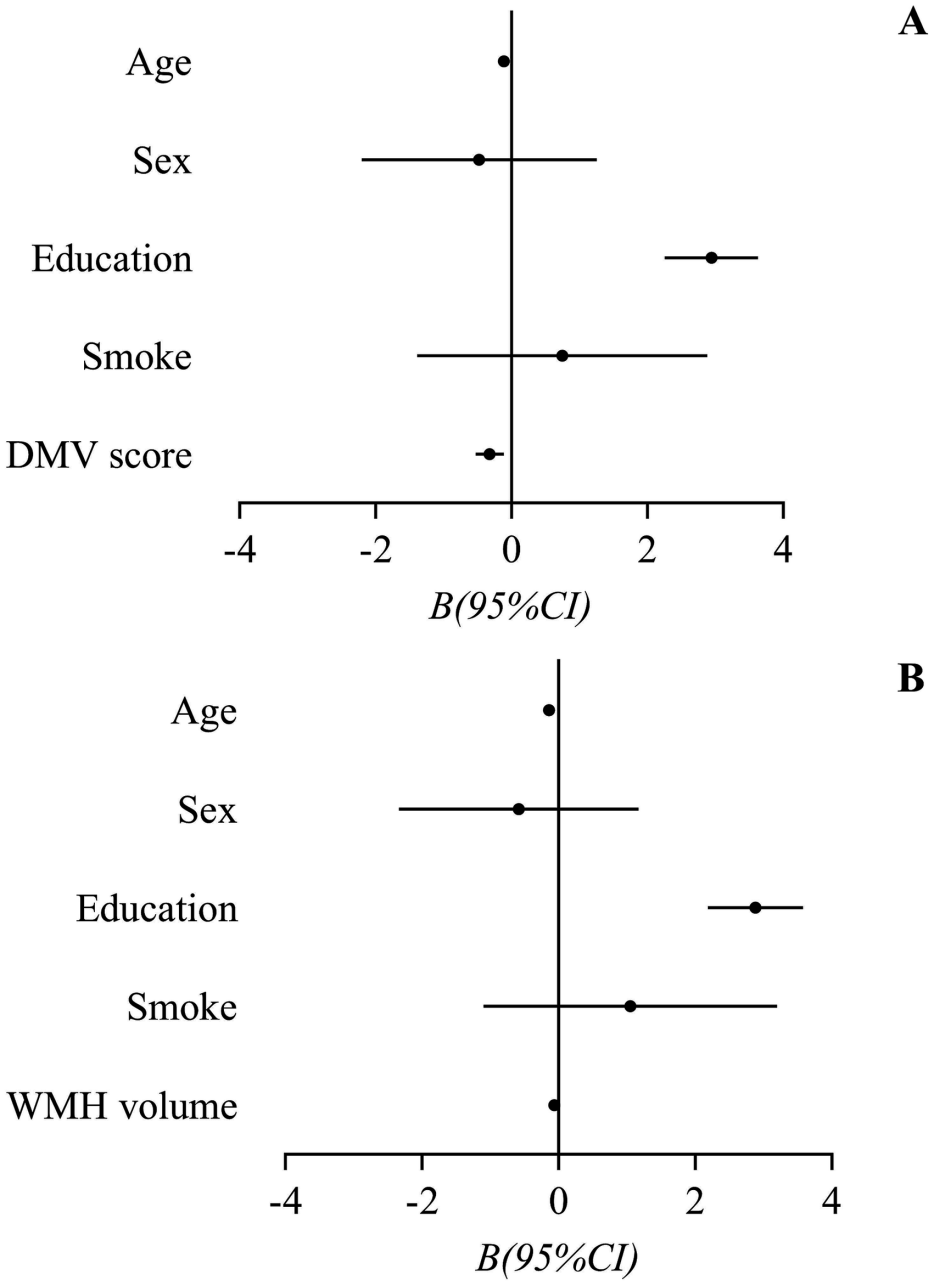
Forest plots of the association between risk factors and Montreal Cognitive Assessment score. **(A)** Regression model incorporating DMV1 score demonstrated significant associations with MoCA score for age, and DMV score, indicating that higher DMV scores correlated with poorer cognitive performance. **(B)** Regression model incorporating WMH volume revealed significant associations with MoCA score for age, education level, and WMH volume, suggesting that increased WMH burden was linked to lower cognitive scores. DMV, deep medullary vein; MoCA, Montreal Cognitive Assessment.

Due to DMV score and WMH volume were highly correlated (*r* = 0.45), two regression models were employed to investigate the association between risk factors and MoCA score. The regression model, which included DMV score, demonstrated that age (*p* = 0.06), the level of education (*p* < 0.001) and DMV score (*p* = 0.003) were significantly associated with MoCA score, suggesting a higher DMV score was related to a lower MoCA score. The regression model with WMH volume included showed that age (*p* < 0.001), the level of education (*p* < 0.001) and WMH volume (*p* = 0.022) had significance. The results of the regressions analysis examining the association between risk factors and MoCA score were showed in Table 4, and the forest plots were presented in Figure 2.

**Table 4:** Multivariate linear regressions analysis of the association between risk factors and MoCA score.

Variable	*β*	SD	*t*	95% CI	*P*
Model 1 (featuring DMV score)					
Age	−0.106	0.038	−2.797	−0.180, −0.031	0.006
Gender	−0.473	0.876	−0.540	−2.203, 1.257	0.590
Education	2.944	0.347	8.475	2.258, 3.630	0.000
Smokers/ex-smokers	0.753	1.080	0.697	−1.381, 2.886	0.487
DMV score	−0.319	0.106	−3.001	−0.528, −0.109	0.003
Model 2 (featuring WMH volume)					
Age	−0.134	0.037	−3.658	−0.206, −0.061	0.000
Gender	−0.581	0.889	−0.653	−2.336, 1.175	0.515
Education	2.885	0.352	8.188	2.190, 3.581	0.000
Smokers/ex-smokers	1.053	1.087	0.969	−1.093, 3.199	0.334
WMH volume	−0.057	0.025	−2.306	−0.107, −0.008	0.022

CI, confidence interval; DMV, deep medullary vein; OR, odds ratio; SD, standard deviation; WMH, white matter hyperintensities.

### 3.3 Path analysis

**Figure 3:**
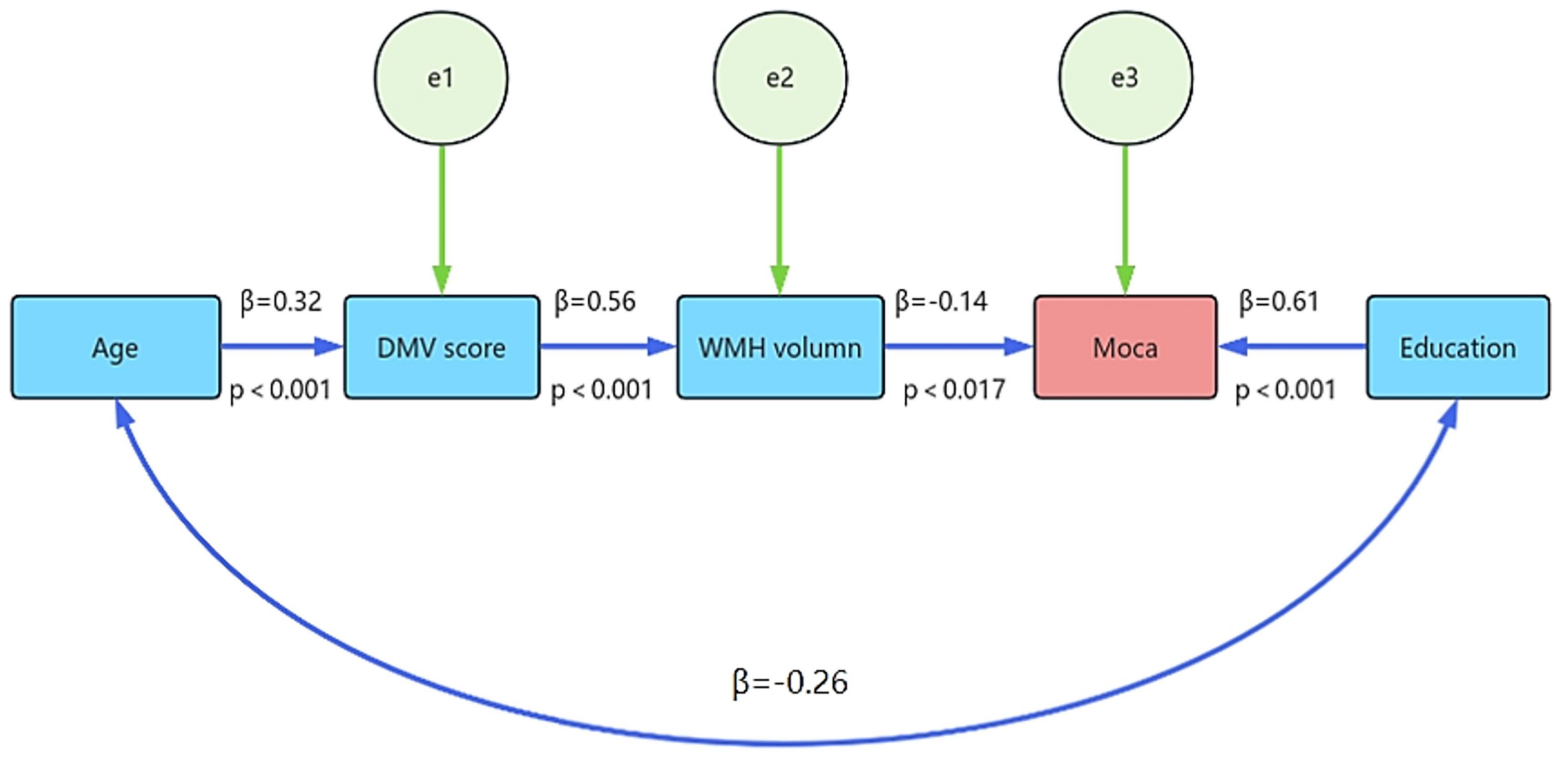
Path analysis of DMV-MoCA association: WMH volume as partial mediator. Path analysis illustrating the DMV score exerted a significant influence on the reduction of the MoCA score, with the WMH volume serving as a partial mediator in this relationship. DMV, deep medullary vein; MoCA, Montreal Cognitive Assessment.

To establish an SEM, variables that exhibited a *p*-value less than 0.05 in the multilinear analysis, including age, education, DMV score, and WMH volume, were selected. The model fit indices indicated good fitness with the following values: chi-square statistic χ^2^ = 20.348, χ^2^/df = 4.070, RMSEA = 0.134, and GFI = 0.954. All indices showed acceptably. Our path analysis demonstrates a plausible mediation pathway linking DMV integrity to cognitive performance (assessed by MoCA) WMH burden. Age exhibited a strong positive association with DMV score (*β* = 0.32, *p* < 0.001), which in turn predicted greater WMH volume (*β* = 0.56, *p* < 0.001). Elevated WMH load was independently associated with lower MoCA scores (*β* = −0.14, *p* = 0.017), even after adjusting for education level (*β* = 0.61, *p* < 0.001). These findings suggest that DMV disruption may contribute to cognitive decline partially via its intermediary effect on WMH accumulation in CSVD (Figure 3).

## 4 Discussion

In our study, we found higher DMV score (indicating more severe DMV disruption) was significantly associated with higher WMH volume, as well as higher number of CMB and LI, which were typical features of CSVD. Additionally, we observed a negative correlation between DMV score and MoCA score, indicating that more severe DMV disruption is associated with worse cognitive performance. A path analysis model revealed that the DMV score exerted an impact on the MoCA score, partially mediated by the WMH volume.

According to a previous study, a higher DMV score was found to indicate more severe disruption of the DMV (21). Therefore, the level of disruption in the DMV can serve as an imaging biomarker for assessing the severity of CSVD. Our findings are consistent with previous studies (16–18). However, compared to previous studies that relied on semi-quantitative assessments, this study employed quantitative measurement methods CSVD imaging markers using an AI neuroimaging system, which improves the reliability of findings.

The mechanism of the relationship between DMV damage and WMH volume increase is still unclear. The damage of DMV may lead to drainage obstruction of lymphatic fluid in the perivascular space, which in turn leads to the accumulation of toxic metabolic byproducts (22) and secondary inflammatory reaction of white matter myelin (23), which might result in damage of white matter. Furthermore, DMV damage leads to increased vascular resistance, resulting in decreased blood flow to the deep white matter (13, 24), causing hypoperfusion in the deep white matter, which is believed to be associated with the occurrence and progression of WMH (25). Additionally, the imbalance of venous system may contribute to the infiltration of inflammatory factors, vascular remodeling, endothelial cell apoptosis (12) and cerebral venous congestion (26), ultimately leading to the disruption of the blood–brain barrier (BBB), and the disruption of BBB is deemed to be one of the pathophysiological mechanisms of CSVD (27). One previous study discovered that BBB permeability increased in the perilesional zones, specifically in areas close to WMH (28). Another prospective study revealed that the baseline leakage of BBB was associated with the 2-year change in parenchymal diffusivity in the perilesional zone of WMH (29). These findings support the hypothesis that BBB impairment might play a critical role in the subsequent white matter degeneration. Therefore, it can be speculated that BBB disruption is one of the potential intermediate links between DMV and WMH.

The impairment to the DMV has been demonstrated to result in the disruption of BBB (12, 13, 26), which has also been identified as a contributing factor to the development of LI and CMB. A study employing gadolinium contrast-enhanced MRI to assess BBB permeability found that patients with LI exhibited higher BBB permeability compared to individuals with cortical ischemic stroke (30). Another case–control study demonstrated that increased BBB permeability occurred in patients with CSVD, including LI, but not in healthy control individuals (31). This finding suggests that BBB dysfunction is associated with the development of LI. A cohort study with 1,039 patients found that patients with a higher number of CMB were more likely to exhibit higher cerebrospinal fluid/serum albumin ratios, indicating blood–brain barrier dysfunction (32). The heightened BBB permeability leads to erythrocyte exudation from small cerebral vessels, which is the hallmark of CMB (33). In summary, the disruption of BBBs may also serve as an intermediate link between DMV and the development of LI and CMB.

The present study demonstrated a negative correlation between the DMV score and the MoCA score, with the latter being adjusted for age, level of education, gender, and history of smoking. This suggests that disruption of the DMV is associated with decreased cognitive performance in patients. This finding is consistent with that of some previous research (34, 35). The following hypotheses are proposed for consideration: As demonstrated in the present study, the DMV has been shown to be associated with the severity of CSVD. Numerous research studies have identified CSVD as a critical risk factor for cognitive impairment (36–38). While the cross-sectional data does not allow for confirmation of causality, the path analysis indicates that the substantial DMV-WMH association suggests that DMV impairment likely occurs prior to WMH development in the CSVD cascade. However, the absence of longitudinal WMH trajectory data precludes definitive conclusions about whether DMV damage independently affects cognition prior to the emergence of overt WMH. Further research employing serial neuroimaging techniques is necessary to elucidate the temporal sequence and potential direct effects of DMV disruption on pre-symptomatic cognitive changes. Secondly, according to the findings of a preceding study, the disruption of the DMV has been demonstrated to be associated with brain atrophy, particularly in the middle and inferior temporal lobes and the hippocampus, which play a pivotal role in cognitive function (39). This suggests that the disruption of the DMV may lead to brain atrophy, consequently resulting in cognitive impairment. Thirdly, the decreased distribution or stenosis of venules leads to impedance of the efflux of perivenous glymphatic fluid and inadequate drainage of glymphatic clearance, resulting in toxic accumulation and structural injury in the cortex (40, 41). Fourthly, disruption of the BBB, which is related to damage of the DMV (12, 13, 26), will then lead to cognitive decline (42, 43).

In the present study, no correlation was identified between the number of CMB and LI and the MoCA score. However, it is plausible that the distribution of CMB and LI may have a more significant impact on cognitive impairment than the mere count of these markers. This hypothesis is corroborated by extant research demonstrating a correlation between strictly lobar CMB, but not deep or infratentorial CMB, and cognitive function decline (37). In the present study, a correlation was observed between the level of SCR and WMH volume. A body of research has previously demonstrated a correlation between chronic kidney disease (CKD) and the severity of WMH (48, 49). However, further studies are required to elucidate the relationship between kidney function and WMH, as well as the potential mechanisms underlying this association.

The present study was characterized by several notable strengths. Firstly, to the best of our knowledge, it was one of the few studies that specifically investigated the correlation between DMV score and CSVD imaging markers using a quantitative neuroimage analytical tool. Secondly, in comparison with previous studies of a similar nature, our research study comprised a more substantial sample size. Thirdly, we conducted a rigorous path analysis to delve into the potential mediating influence of DMV and WMH volume on the decline of MoCA score. However, it is imperative to interpret the findings within the context of several limitations. Firstly, the evaluation of additional CSVD imaging markers, including enlarged perivascular space, brain atrophy, and cortical cerebral microinfarct, was precluded by the limitations of the AccuBrain@ software. Secondly, the absence of a validated quantitative method for DMV necessitated the use of a semi-quantitative assessment in this study, potentially limiting measurement precision. Thirdly, due to the cross-sectional nature of the study, longitudinal data were not available. Fourthly, participant enrollment was not strictly consecutive, which may have introduced selection bias. Although a systematic screening protocol was implemented, the potential for this bias should be considered when interpreting the results, as it may affect the generalizability of our findings.

## 5 Conclusion

In summary, the present findings demonstrate that the DMV score is associated with the severity of WMH, CMB, and LI, as well as cognitive performance decline. This finding suggests a potential link between cerebral venule damage and the development of CSVD, as well as cognitive impairment. However, further longitudinal investigations with larger sample sizes are necessary to confirm these observations.

## Data Availability

The raw data supporting the conclusions of this article will be made available by the authors, without undue reservation.
