# Supplementary figures and images for: Deep medullary veins disruption in cerebral small vessel disease: links to AI-quantified lesions and cognitive decline
Source: Front Neurol. 2025 Oct 20;16:1647684. doi: 10.3389/fneur.2025.1647684 (PMC12580129; doi:10.3389/fneur.2025.1647684)

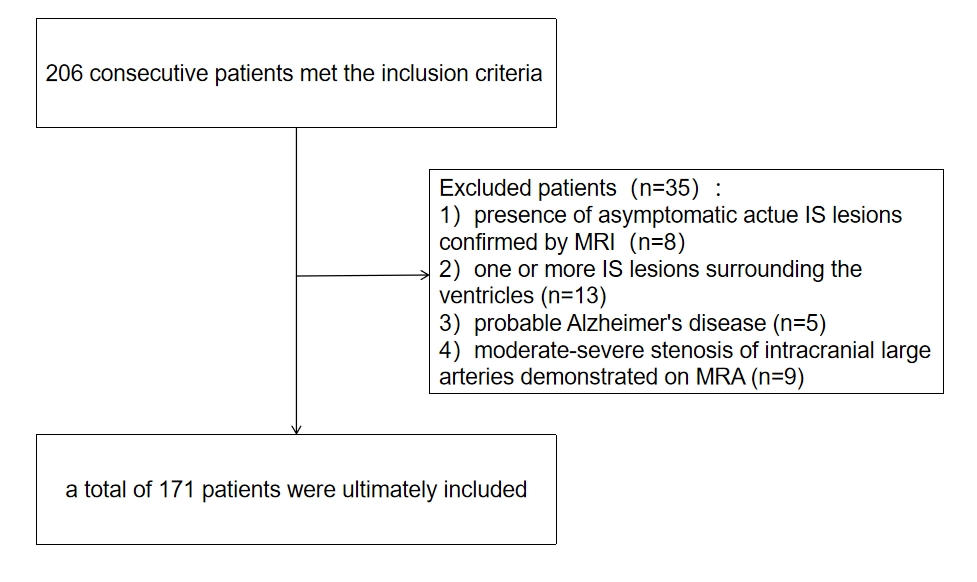

Supplement: Supplementary file 1 [file Image_1.JPEG]
