# Supplementary material for: Deep medullary veins disruption in cerebral small vessel disease: links to AI-quantified lesions and cognitive decline
Source: Front Neurol. 2025 Oct 20;16:1647684. doi: 10.3389/fneur.2025.1647684 (PMC12580129; doi:10.3389/fneur.2025.1647684)
Supplement: Supplementary file 2 [file Table_1.docx]

**Supplementary Table 1**

Univariate ordinal logistic regression analysis of the association between risk factors and WMH volume.

| Variable | β | *P* | *OR* | 95%CI |
| --- | --- | --- | --- | --- |
| Age | 0.027 | 0.060 | 1.027 | 0.999-1.056 |
| Gender | 0.179 | 0.515 | 1.197 | 0.698-2.052 |
| Hypertension | 0.052 | 0.862 | 1.054 | 0.584-1.900 |
| Diabetes | -0.526 | 0.079 | 0.591 | 0.328-1.064 |
| Smokers/ex-smokers | -0.096 | 0.783 | 0.909 | 0.461-1.793 |
| serum creatinine | 0.016 | 0.010 | 1.016 | 1.004-1.029 |
| Uric Acid | 0.000 | 0.765 | 1.000 | 0.998-1.003 |
| TCH | -0.003 | 0.222 | 0.997 | 0.993-1.002 |
| LDL-C | -0.132 | 0.364 | 0.876 | 0.658-1.166 |
| Hcy | 0.057 | 0.117 | 1.059 | 0.986-1.137 |
| HbA1c | 0.077 | 0.547 | 1.080 | 0.841-1.388 |
| DMV score | 0.280 | 0.000 | 1.323 | 1.208-1.449 |

Notes:

CI=confidence interval; DMV=deep medullary vein; HbA1c=glycated hemoglobin, type A1c; Hcy=homocysteine; LDL-C=low density lipoprotein cholesterin；OR=odds ratio; WMH=white matter hyperintensities.
