# Supplementary material for: Deep medullary veins disruption in cerebral small vessel disease: links to AI-quantified lesions and cognitive decline
Source: Front Neurol. 2025 Oct 20;16:1647684. doi: 10.3389/fneur.2025.1647684 (PMC12580129; doi:10.3389/fneur.2025.1647684)
Supplement: Supplementary file 3 [file Table_2.docx]

**Supplementary Table 2**

Univariate ordinal logistic regression analysis of the association between risk factors and CMB number.

| Variable | β | *P* | *OR* | 95%CI |
| --- | --- | --- | --- | --- |
| Age | -0.024 | 0.143 | 0.977 | 0.946-1.008 |
| Gender | -0.018 | 0.954 | 0.982 | 0.527-1.830 |
| Hypertension | 0.087 | 0.804 | 1.090 | 0.550-2.161 |
| Diabetes | 0.063 | 0.853 | 1.065 | 0.544-2.085 |
| Smokers/ex-smokers | -0.221 | 0.589 | 0.802 | 0.360-1.786 |
| serum creatinine | 0.008 | 0.243 | 1.008 | 0.995-1.021 |
| Uric Acid | -0.001 | 0.651 | 0.999 | 0.996-1.002 |
| TCH | -0.155 | 0.205 | 0.856 | 0.674-1.088 |
| LDL-C | -0.293 | 0.101 | 0.746 | 0.526-1.059 |
| Hcy | 0.051 | 0.217 | 1.053 | 0.970-1.142 |
| HbA1c | -0.161 | 0.334 | 0.851 | 0.613-1.180 |
| DMV score | 0.112 | 0.014 | 1.119 | 1.023-1.224 |

Notes:

CI=confidence interval; CMB=cerebral microbleed; DMV=deep medullary vein; HbA1c=glycated hemoglobin, type A1c; Hcy=homocysteine; LDL-C=low density lipoprotein cholesterin；OR=odds ratio.
