# Supplementary material for: Deep medullary veins disruption in cerebral small vessel disease: links to AI-quantified lesions and cognitive decline
Source: Front Neurol. 2025 Oct 20;16:1647684. doi: 10.3389/fneur.2025.1647684 (PMC12580129; doi:10.3389/fneur.2025.1647684)
Supplement: Supplementary file 4 [file Table_3.docx]

**Supplementary Table 3**

Univariate ordinal logistic regression analysis of the association between risk factors and LI number.

| Variable | β | *P* | *OR* | 95%CI |
| --- | --- | --- | --- | --- |
| Age | -0.008 | 0.562 | 0.992 | 0.966-1.019 |
| Gender | 0.254 | 0.358 | 1.289 | 0.750-2.216 |
| Hypertension | -0.198 | 0.512 | 0.820 | 0.453-1.483 |
| Diabetes | -0.088 | 0.767 | 0.915 | 0.510-1.642 |
| Smokers/ex-smokers | 0.018 | 0.959 | 1.018 | 0.515-2.012 |
| serum creatinine | 0.003 | 0.586 | 1.003 | 0.992-1.014 |
| Uric Acid | -0.001 | 0.365 | 0.999 | 0.996-1.001 |
| TCH | 0.000 | 0.824 | 1.000 | 0.996-1.004 |
| LDL-C | -0.002 | 0.802 | 0.998 | 0.986-1.011 |
| Hcy | 0.005 | 0.888 | 1.005 | 0.937-1.078 |
| HbA1c | -0.132 | 0.313 | 0.876 | 0.678-1.133 |
| DMV score | 0.132 | 0.001 | 1.141 | 1.054-1.234 |

Notes:

CI=confidence interval; DMV=deep medullary vein; HbA1c=glycated hemoglobin, type A1c; Hcy=homocysteine; LDL-C=low density lipoprotein cholesterin；LI=lacunar infarction; OR=odds ratio.
