# Supplementary material for: Deep medullary veins disruption in cerebral small vessel disease: links to AI-quantified lesions and cognitive decline
Source: Front Neurol. 2025 Oct 20;16:1647684. doi: 10.3389/fneur.2025.1647684 (PMC12580129; doi:10.3389/fneur.2025.1647684)
Supplement: Supplementary file 5 [file Table_4.docx]

**Supplementary Table 4**

| Variable | β | SD | t | 95%CI | *P* |
| --- | --- | --- | --- | --- | --- |
| Age | -0.229 | 0.044 | -5.220 | -0.315,-0.142 | 0.000 |
| Gender | -3.425 | 0.924 | -3.707 | -5.248,-1.601 | 0.000 |
| education | 3.422 | 0.335 | 10.219 | 2.761,4.084 | 0.000 |
| Hypertension | 1.075 | 1.044 | 1.030 | -0.986,3.136 | 0.305 |
| Diabetes | -1.440 | 1.030 | -1.399 | -3.473,0.593 | 0.164 |
| Smokers/ex-smokers | 3.396 | 1.192 | 2.850 | 1.043,5.748 | 0.005 |
| serum creatinine | 0.034 | 0.019 | 1.762 | -0.004,0.073 | 0.080 |
| Uric Acid | 0.009 | 0.005 | 1.941 | 0.000,0.018 | 0.054 |
| TCH | -0.011 | 0.007 | -1.590 | -0.025,0.003 | 0.114 |
| LDL-C | 0.003 | 0.022 | 0.113 | -0.042,0.047 | 0.910 |
| Hcy | 0.047 | 0.126 | 0.368 | -0.203,0.297 | 0.713 |
| HbA1c | -0.143 | 0.448 | -0.319 | -1.027,0.741 | 0.750 |
| WMH volume | -0.079 | 0.033 | -2.438 | -0.143,-0.015 | 0.016 |
| CMB number | 0.083 | 0.088 | 0.941 | -0.091,0.257 | 0.348 |
| LI number | -0.013 | 0.083 | -0.158 | -0.178,0.152 | 0.875 |
| DMV score | -0.511 | 0.130 | -3.940 | -0.767,-0.255 | 0.000 |

Univariate linear regression analysis of the association between risk factors and MoCA score.

Notes:

CI=confidence interval; CMB=cerebral microbleed; DMV=deep medullary vein; HbA1c=glycated hemoglobin, type A1c; Hcy=homocysteine; LDL-C=low density lipoprotein cholesterin；LI=lacunar infarction；MoCA=Montreal Cognitive Assessment；OR=odds ratio; SD=standard deviation; WMH=white matter hyperintensities.
